# Induction of transplantation tolerance converts potential effector T cells into graft-protective regulatory T cells

**DOI:** 10.1002/eji.201040509

**Published:** 2010-12-08

**Authors:** Ross S Francis, Gang Feng, Thanyalak Tha-In, Ian S Lyons, Kathryn J Wood, Andrew Bushell

**Affiliations:** Transplant Research Immunology Group, Nuffield Department of Surgery, University of Oxford John Radcliffe HospitalOxford, UK

**Keywords:** Transplantation tolerance, Treg

## Abstract

Naturally occurring FOXP3^+^CD4^+^ Treg have a crucial role in self-tolerance. The ability to generate similar populations against alloantigens offers the possibility of preventing transplant rejection without indefinite global immunosuppression. Exposure of mice to donor alloantigens combined with anti-CD4 antibody induces operational tolerance to cardiac allografts, and generates Treg that prevent skin and islet allograft rejection in adoptive transfer models. If protocols that generate Treg *in vivo* are to be developed in the clinical setting it will be important to know the origin of the Treg population and the mechanisms responsible for their generation. In this study, we demonstrate that graft-protective Treg arise *in vivo* both from naturally occurring FOXP3^+^CD4^+^ Treg and from non-regulatory FOXP3^−^CD4^+^ cells. Importantly, tolerance induction also inhibits CD4^+^ effector cell priming and T cells from tolerant mice have impaired effector function *in vitro*. Thus, adaptive tolerance induction shapes the immune response to alloantigen by converting potential effector cells into graft-protective Treg and by expanding alloreactive naturally occurring Treg. In relation to clinical tolerance induction, the data indicate that while the generation of alloreactive Treg may be critical for long-term allograft survival without chronic immunosuppression, successful protocols will also require strategies that target potential effector cells.

## Introduction

Current therapy for patients undergoing transplantation relies upon indefinite non-specific immunosuppression to prevent rejection. Although modern immunosuppressive regimens have dramatically improved early transplant outcomes 1, there has been less impact on the rate of late graft loss 2. Furthermore, chronic immunosuppression exposes transplant recipients to drug toxicity and an increased risk of malignancy 3 and infection 4. A growing understanding of the intrinsic immunological mechanisms that control autoimmunity has led to the concept that targeted immune-manipulation with agents such as anti-CD3 5 or anti-CD52 antibodies 6, 7 may promote regulation of donor-directed immune responses, thus leading to improved graft outcomes while preserving protective immunity.

Naturally occurring CD4^+^ Treg that develop in the thymus under the control of the transcription factor *foxp3* 8–10 have a critical role in peripheral immune homeostasis and lack of Treg, due to congenital deficiency of *foxp3* 11, 12 or selective depletion of *foxp3*-expressing cells, 13, 14 results in fatal autoimmune disease. In addition to controlling auto-reactive cells, Treg have also been shown to suppress immune responses to foreign antigens in the context of pregnancy 15, malignancy 16, infection 17 and transplantation 18. In several experimental models, Treg are essential for the induction and maintenance of transplantation tolerance 19–23 and the fact that regulation invariably depends on TCR ligation provides the possibility of using specific antigens to induce regulatory function in defined situations. We have shown that pre-treatment of naïve mice with a DST (donor-specific transfusion) combined with anti-CD4 mAb generates CD25^+^CD4^+^ T cells that prevent rejection of skin allografts by a defined effector population in lymphopenic adoptive transfer recipients 21, 24. Importantly, this tolerance induction protocol also leads to indefinite cardiac allograft survival in immunologically intact recipients 25.

Successful translation of tolerance induction for potential therapeutic use will depend on an understanding of how alloantigen-reactive Treg are generated. In this study we provide evidence that graft-protective Treg can arise in vivo both by expansion of endogenous, naturally occurring FOXP3^+^CD4^+^ regulatory cells and by conversion of FOXP3^−^CD4^+^ non-regulatory precursors. Moreover, we also demonstrate that tolerance induction shapes the immune response to alloantigen by inhibiting the priming of IFN-γ-secreting CD4^+^ effector cells, resulting ultimately in impaired effector function.

## Results

### Tolerance induction with anti-CD4+DST is dependent on CD25^+^ Treg

**Figure 1:**
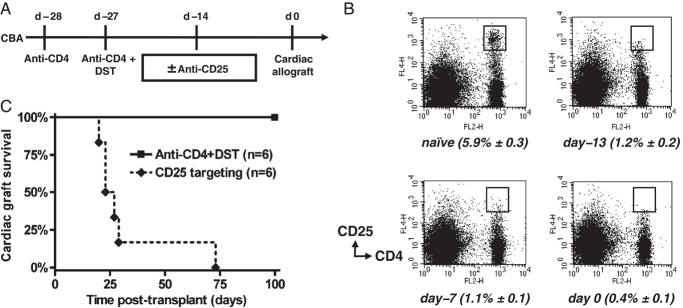
Tolerance induction with anti-CD4+DST is dependent on CD25^+^ Treg. (A) CBA mice (H2^k^) were pre-treated with 200 μg of YTS177 (anti-CD4 mAb) i.v. on days 28 and 27 together with 250 μL whole H2^b^ blood (DST) on day 27. On day 14, mice in the CD25 targeting group received 1 mg of PC61 (anti-CD25 mAb) i.v. On day 0, donor-type (H2^b^) vascularised heterotopic cardiac allografts were transplanted. (B) Representative plots (gated on viable lymphocytes) showing depletion of CD25^+^ T cells. Figures show percentage of CD25^+^CD4^+^ cells in the region indicated (mean±SEM, *n*=3 mice per time point). (C) Cardiac allograft survival for mice in (A).

CBA (H2^k^) mice pre-treated with DST and non-depleting anti-CD4 mAb (YTS177.9) on days 28 and 27 accept donor cardiac allografts on day 0 without further immunotherapy 25. To test the hypothesis that heart allograft survival in immunocompetent primary recipients is dependent on induced CD25^+^CD4^+^ Treg, CBA mice were pre-treated with anti-CD4+H2^b^ DST on days 28 and 27, ±anti-CD25 mAb (PC61) on day 14 and transplanted with H2^b^ cardiac allografts on day 0 (Fig. 1A). Administration of anti-CD25 mAb results in >90% depletion of CD25^+^CD4^+^ cells at the time of transplantation (day 0, Fig. 1B) and at this time point is cleared from the circulation (data not shown), thus depleting CD25^+^ Treg without impairing effector T-cell responses driven by the graft.

Tolerised mice accepted donor grafts indefinitely (*n*=6, MST (median survival time)>100 days, Fig. 1C) but in contrast, mice that also received depleting anti-CD25 mAb rejected their grafts acutely (*n*=6, MST 25 days) confirming that CD25^+^ Treg have a non-redundant role in allograft acceptance in this model, a finding supported by recent work demonstrating that ligation of glucocorticoid-induced TNFR-related protein (GITR) at the time of transplantation also prevents graft acceptance 20.

### Tolerant mice are enriched for graft-protective Treg

**Figure 2:**
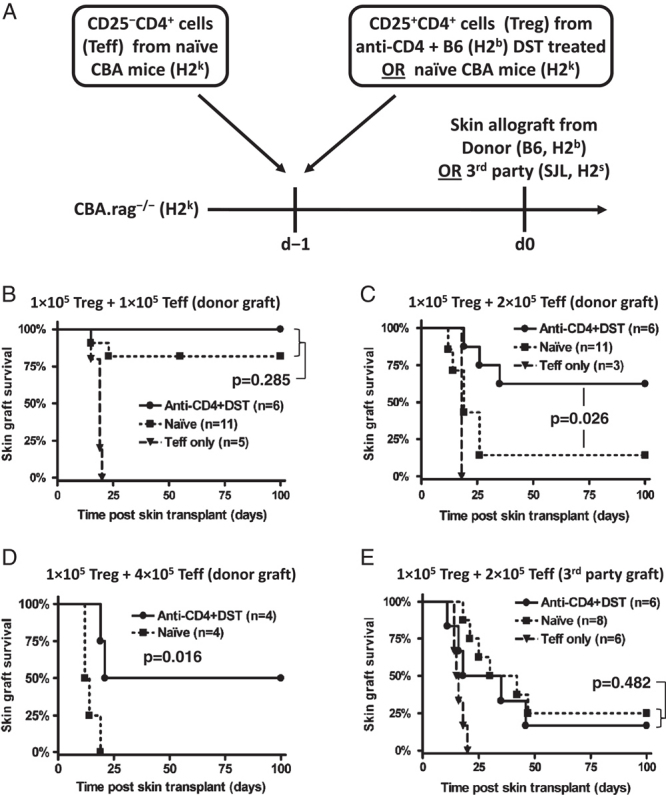
Tolerant mice are enriched for graft-protective Treg. (A) T- and B–cell-deficient CBA.rag^−/−^ mice (H2^k^) were reconstituted with CD25^−^CD4^+^ cells (Teff) purified from un-manipulated CBA mice and CD25^+^CD4^+^ cells (Treg) purified from either anti-CD4 + H2^b^ DST treated or naïve un-manipulated mice on day 1. On day 0, H2^b^ (donor) or H2^s^ (third party) skin allografts were transplanted. Data are from four independent experiments. Survival was compared using the Log-rank test. (B) Donor graft survival after reconstitution with 1×10^5^ Treg+1×10^5^ Teff. (C) Donor graft survival after reconstitution with 1×10^5^ Treg+2×10^5^ Teff. (D) Donor graft survival after reconstitution with 1×10^5^ Treg+4×10^5^ Teff. (E) Third-party graft survival after reconstitution with 1×10^5^ Treg+2×10^5^ Teff.

CD25^+^CD4^+^ cells from tolerant mice prevent donor skin graft rejection mediated by naïve CD4^+^ effector cells after adoptive transfer into lymphopenic recipients 21, 24 but work by other groups using similar adoptive transfer models has shown that naïve CD25^+^CD4^+^ cells can also prevent rejection when transferred at a high Treg:Teff ratio 26, 27. However, the fact that naïve mice reject cardiac allografts within 8 days 25 whereas mice tolerised by the anti-CD4+DST protocol accept their grafts long-term in a manner dependent on Treg (Fig. 1) indicates a quantitative or qualitative difference between tolerised Treg and their naïve counterparts. This prompted us to compare directly the function of CD25^+^CD4^+^ cells from tolerant and naïve unmanipulated mice in the same adoptive transfer system. Immunodeficient CBA.rag (H2^k^) mice were reconstituted with naïve syngeneic CD25^−^CD4^+^ cells as an effector population together with CD25^+^CD4^+^ cells from tolerant or unmanipulated CBA mice (Fig. 2A). After 1 day, full-thickness H2^b^ skin grafts were transplanted. At a 1:1 Treg:Teff ratio, CD25^+^CD4^+^ cells from either naïve or tolerised mice prevented rejection (Fig. 2B). However, when the number of Treg was kept constant but the number of effector T cells increased, a distinct difference emerged in that CD25^+^CD4^+^ cells from anti-CD4+DST-treated mice were significantly more effective than CD25^+^CD4^+^ cells from naïve mice (Fig. 2C and D). To ask whether this effect was donor-specific, experiments were performed using third party skin grafts from SJL mice (H2^s^). As shown in Fig. 2E, there was no significant difference in the ability of CD25^+^CD4^+^ cells from naïve or tolerised mice to prevent SJL skin graft rejection. Thus, compared with unmanipulated mice, the CD25^+^CD4^+^ T-cell pool from mice tolerised to donor alloantigen is enriched significantly for cells with the ability to control the rejection of donor-strain allografts.

To ask whether this in vivo difference in Treg function could be modelled in vitro, a proliferation suppression assay was developed in which CFSE-labelled CD25^−^CD4^+^ responder cells from CBK mice (H2^k^+transgenic K^b^) were cultured with graded numbers of CD25^+^CD4^+^ cells (Treg) from either naïve or anti-CD4+DST-treated CBA mice (H2^k^) (Supporting Information Fig. 1A). Irradiated splenocytes from CBA×B10 F1 mice (H2^k+b^) were used as stimulators to provide direct stimulation via I-A^b^ while also providing an exclusion gate in the proliferation analysis. As shown in Supporting Information Fig. 1B, at a 1:1 ratio, both naïve and tolerised Treg suppressed efficiently the proliferation of CD25^−^CD4^+^ responder cells but unlike the in vivo situation (Fig. 2), a titration of the Treg:responder ratio did not reveal a difference between the two populations. Although its simplicity makes the MLR an attractive assay with which to examine in vivo responses, the overall clinical experience has been that mixed lymphocyte responses do not provide a reliable prediction or reflection of graft outcome 28 and, indeed, we have previously noted a clear disparity between the in vivo and in vitro suppressive capacity of CD25^+^CD4^+^ Treg 29. These observations highlight the limitations of the MLR to model in vivo responses and stress the importance of functional in vivo models.

### GITR^−^CD25^−^CD4^+^ cells are depleted of naturally occurring Treg

**Figure 3:**
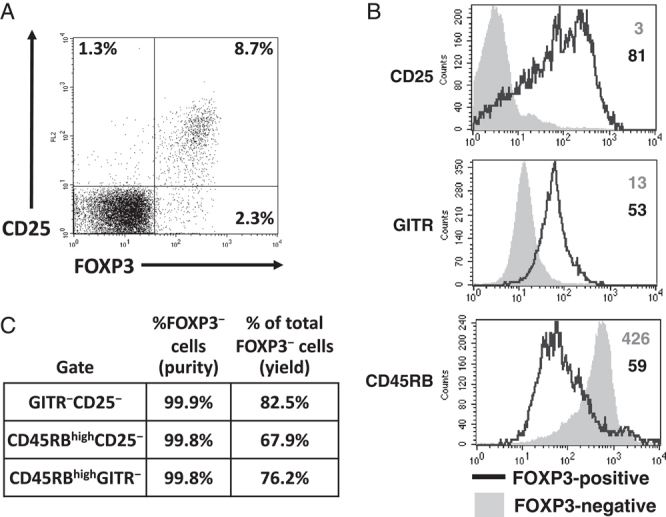
Correlation of phenotypic markers with FOXP3 expression. Splenocytes from naïve CBA mice were stained for CD4, cell surface markers and FOXP3. Histograms are gated on live CD4^+^ cells, and are representative of three independent experiments. (A) Expression of CD25 and FOXP3 in gated viable CD4^+^ cells. (B) Markers that correlated with FOXP3 expression. Figures indicate median fluorescence intensities for FOXP3^−^ and FOXP3^+^ populations. (C) Predicted yield and purity of FOXP3 cells based on the pairs of markers shown.

The observation that the precursor frequency of alloreactive cells within naturally occurring Treg is similar to that in potential T effector cells 30 combined with the fact naïve CD25^+^CD4^+^ cells can prevent allograft rejection at high Treg:Teff ratios (Fig. 2) suggests that pre-existing, endogenous Treg appear to make a significant contribution to graft protection. However, naïve CD4^+^ T cells can undergo conversion into functional Treg following antigen encounter in the peripheral immune system 31, 32, indicating that conversion can also play a significant role. We have previously shown that when purified CD25^−^CD4^+^ T cells are transferred into immunodeficient mice, these can be driven to become graft-protective Treg by the anti-CD4+DST tolerising protocol, suggesting that non-Treg precursors can be converted to alloreactive Treg *in vivo* 24. However, CD25 is an imperfect marker for Treg and recent evidence has identified the transcription factor FOXP3 as a master control gene for naturally occurring Treg development, potentially providing an improved marker for these cells in the mouse 9, 10. The limitations of CD25 for distinguishing Treg from non-Treg is shown by the fact that some 20% of FOXP3^+^ cells are contained within the CD25^−^CD4^+^ population (Fig. 3A). Furthermore, experiments using FOXP3^GFP^-reporter mice have demonstrated that in vitro, GFP^+^CD25^−^CD4^+^ cells suppress polyclonally stimulated naïve CD4^+^ T cells as efficiently as GFP^+^CD25^+^CD4^+^ cells 33. Thus, regulation observed in our previous experiments 24 could have been due to expansion of FOXP3^+^ cells contained within the adoptively transferred CD25^−^CD4^+^ population rather than to de novo generation of Treg from non-regulatory precursors.

We therefore sought a rigorous strategy to purify naïve FOXP3^−^CD4^+^ cells from WT mice in order to assess the importance of non-regulatory cell conversion in allograft tolerance. Although B6 (H2^b^) FOXP3^GFP^-reporter were available to us, we deliberately sought a strategy that would allow us to isolate CD4^+^ T cells devoid of nTreg from CBA (H2^k^) mice to allow direct comparisons to be made with the results of our previous study 24. To identify surrogate markers that might allow flow-purification of FOXP3 negative cells, un-stimulated CBA CD4^+^ T cells were stained for FOXP3 and markers associated with Treg phenotype and function, including CD127, CD25, GITR, CTLA-4, CD62L, CD45RB and CD103. The markers that allowed the most consistent discrimination between FOXP3^+^ and FOXP3^−^ cells were GITR, CD45RB and CD25 (Fig. 3B). The data were then re-analysed using pairs of markers to calculate potential yields and purities from FACS sorting. The highest predicted purity and yield of CD4^+^FOXP3^−^ cells were obtained using a combination of the markers CD4, CD25 and GITR (Fig. 3C).

**Figure 4:**
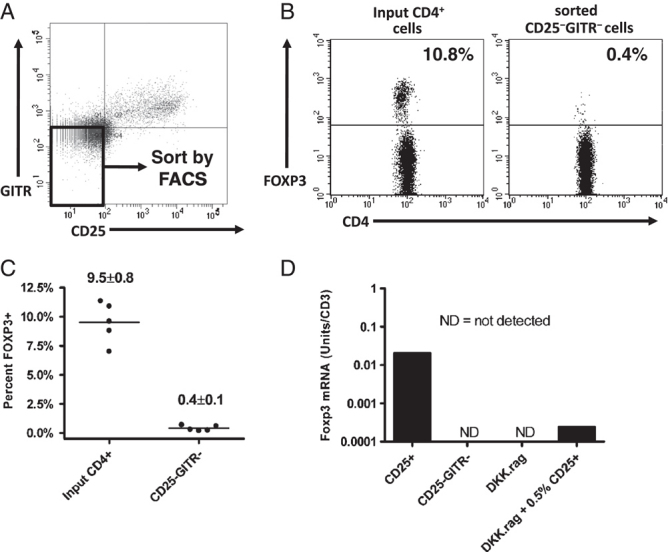
CD25^−^GITR^−^CD4^+^ cells are essentially devoid of nTreg. (A) CBA splenocytes were stained for GITR, CD25 and CD4 and gated CD4^+^ cells with the lowest expression of CD25 and GITR were separated by FACS. (B) Input CD4^+^ and sorted CD25^−^GITR^−^ cells were permeabilised and stained for intracellular FOXP3 (gated on live CD4^+^ cells). (C) Combined results from five independent FACS sorts (mean±SEM). (D) Sorted CD25^−^GITR^−^ cells were analysed for foxp3 mRNA expression by qRT-PCR. Naïve CBA FACS separated CD25^+^ cells were used as a positive control. DKK.rag TCRtg CD4^+^ cells were used as a negative control. In addition, DKK.rag CD4^+^ cells were spiked with 0.5% naïve CBA CD25^+^ cells. Results are normalised against CD3 expression and are representative of three independent experiments.

To validate this strategy for isolating viable cells devoid of naturally occurring Treg, GITR^−^CD25^−^CD4^+^ cells were sorted from naïve CBA mice (Fig. 4A) and the resultant population stained for intracellular FOXP3. Approximately 10% of freshly isolated CD4^+^ cells were FOXP3^+^, but sorted GITR^−^CD25^−^CD4^+^ cells consistently contained ≤0.5% FOXP3^+^ cells (Fig. 4B and C). Indeed, in our hands this strategy was as effective as sorting GFP^−^ cells from FOXP3^GFP^-reporter mice, suggesting the utility of this approach in other non-transgenic mouse strains. As an additional validation step, qRT-PCR was performed on sorted GITR^−^CD25^−^CD4^+^ cells to detect the presence of *foxp3* mRNA. CD4^+^ cells from TCR-transgenic DKK.rag^−/−^ mice, which do not express FOXP3, were used as a negative control. Neither sorted GITR^−^CD25^−^CD4^+^ cells nor DKK.rag^−/−^ cells generated a *foxp3* signal (Fig. 4D). However, *foxp3* mRNA was detected in DKK.rag^−/−^ cells spiked with 0.5% freshly isolated CD25^+^CD4^+^ cells. These data therefore validate this strategy for the isolation of viable populations essentially devoid of naturally occurring Treg. For convenience, this population will be referred to as FOXP3^−^ cells.

### Alloreactive Treg can develop from FOXP3^−^ cells in vivo

**Figure 5:**
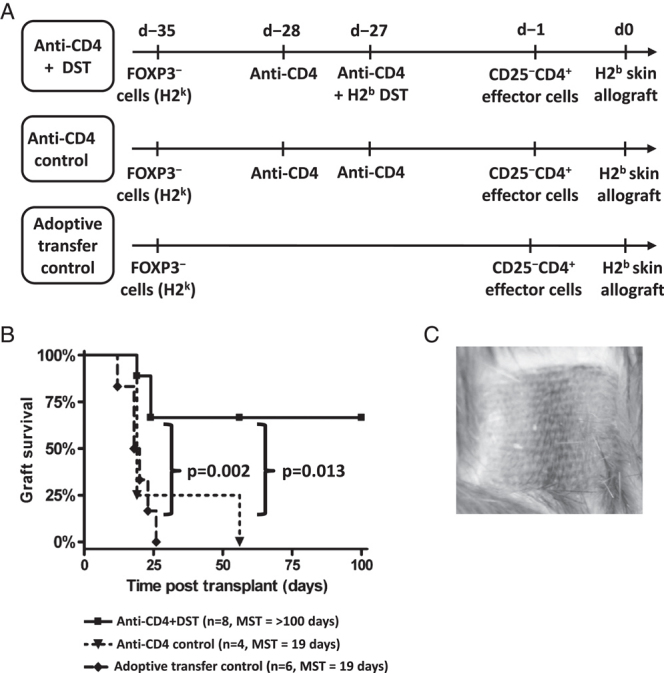
Graft-protective Treg develop from FOXP3^−^ precursors in vivo. (A) CBA.rag mice (H2^k^) were reconstituted with 1×10^6^ sorted naïve CBA (H2^k^) FOXP3 cells on day 35; 200 μg of YTS177 (anti-CD4 mAb) was administered i.v. on days 28 and 27, together with 250 μL whole H2^b^ blood DST on day 27; 1×10^5^ naïve CD25CD4^+^ CBA (H2^k^) effector cells were adoptively transferred on day 1. A full thickness H2^b^ skin allograft was transplanted on day 0. Controls received anti-CD4 mAb without DST (anti-CD4 control) or adoptive transfer of cells alone (adoptive transfer control). Data are from two independent experiments. (B) Skin allograft survival. Survival was compared using the Log-rank test. (C) Representative image of skin graft at day 100 post-transplant.

To ask whether a proven tolerance induction protocol can generate functional Treg from non-regulatory precursors, CBA.rag mice (H2^k^) were reconstituted with 1×10^6^ sorted naïve CBA FOXP3^−^ cells on day 35 (Fig. 5A). Anti-CD4 mAb was administered i.v. on days 28 and 27 and 250 μL whole H2^b^ blood on day 27. On day −1, reconstituted mice additionally received 1×10^5^ naïve syngeneic CD25^−^CD4^+^ T cells to provide effectors (sufficient to reject a skin graft in CBA.rag mice, 24) that had not been exposed to the tolerance induction protocol, making it possible to examine the effects of tolerance induction on Treg generation independently of effects on alloreactive effector cells. Thus, in this system, survival of a full-thickness H2^b^ skin allograft transplanted on day 0 would depend on conversion of FOXP3^−^ precursors into Treg. Control mice received naïve CD25^−^ cells on day −1 and either no pre-treatment or anti-CD4 mAbs alone (Fig. 5A)

As shown in Fig. 5B, adoptive transfer control and anti-CD4 control mice rejected their skin allografts acutely (MST 19 days, *n*=6 and 19 days *n*=4, respectively). In contrast, the majority of mice in the tolerance induction group accepted their grafts long term (MST>100 days, *n*=8) with no signs of graft necrosis (Fig. 5C). Thus, Treg with the capacity to prevent skin allograft rejection can be converted from FOXP3^−^ precursors, implying that naturally occurring Treg are not required for tolerance induction in all situations.

**Figure 6:**
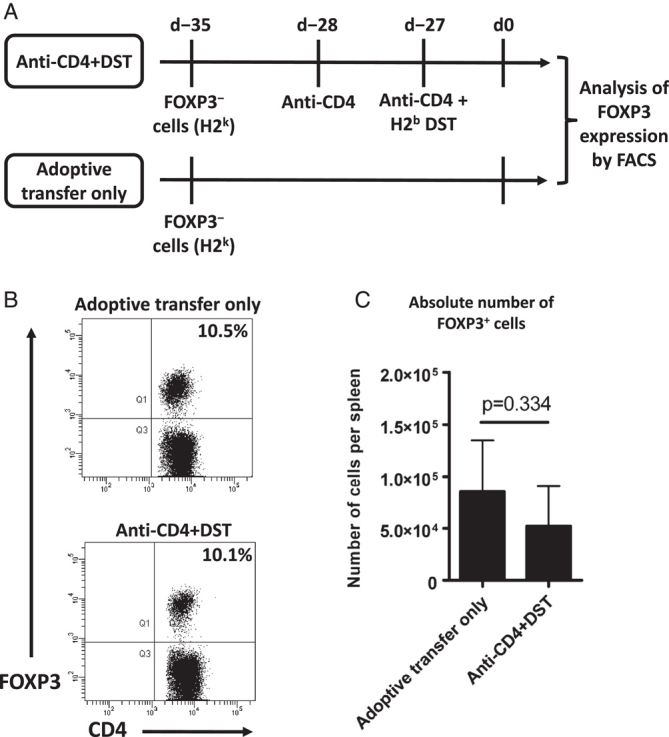
FOXP3 analysis fails to reflect Treg generation from non-Treg precursors. (A) CBA.rag mice (H2^k^) were reconstituted with 1×10^6^ sorted CBA (H2^k^) FOXP3^−^ cells on day 35. The anti-CD4+DST group received 200 μg of YTS177 (anti-CD4) i.v. on days 28 and 27 together with 250 μL whole H2^b^ blood (DST) on day 27. Controls received adoptive transfer of cells alone. On day 0, spleens were harvested and FOXP3 expression analysed by FACS. Representative data are shown from two independent experiments. (B) Proportion of TCR-β^+^CD4^+^ cells expressing FOXP3. (C) Absolute number of FOXP3^+^ T cells per spleen (mean±SD). Statistical analysis using the *t* test.

To test the hypothesis that tolerance induction induces FOXP3 expression in non-regulatory cells, further cohorts of CBA.rag^−/−^ mice (*n*=3–4 per group) were reconstituted with 1×10^6^ FOXP3^−^ cells as above, with or without tolerance induction (Fig. 6A). On day 0, spleens were harvested and FOXP3 expression analysed by FACS. FOXP3^+^ cells were readily detected in both tolerised mice and controls (Fig. 6B), and unexpectedly there was no significant difference in the absolute number of FOXP3^+^ T cells between the two groups (Fig. 6C), indicating that homeostatic proliferation of FOXP3^−^ cells is sufficient to drive FOXP3 expression, consistent with other recent reports 34, 35. Critically, however, only Treg generated following tolerance induction were capable of preventing graft rejection (Fig. 5). These data highlight an important functional distinction between Treg generated in the presence or absence of tolerogenic therapy, and again show the limitations of phenotypic analysis for inferring Treg function, particularly in lymphopenic recipients.

**Figure 7:**
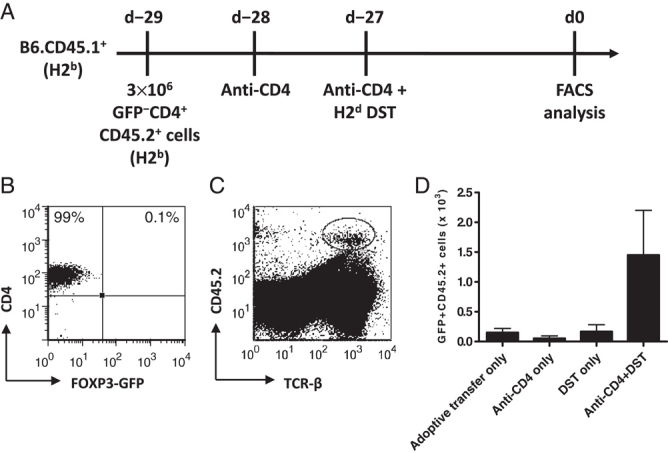
Treg develop from FOXP3^−^ precursors in immunocompetent mice. (A) 3×10^6^ GFP^−^CD4^+^CD45.2^+^ T cells purified from FOXP3-GFP reporter mice by flow cytometry were adoptively transferred into congenic CD45.1^+^ B6 mice on day 29. Recipient mice received 200 μg anti-CD4 mAb (YTS177) on days 28 and 27 with 250 μL DBA2 (H2^d^) blood transfusion (DST) on day 27. Control mice received anti-CD4 mAb alone, DST alone or adoptive transfer of GFP^−^CD4^+^CD45.2^+^ T cells only. On day 0, spleens were harvested and the number of GFP^+^CD4^+^CD45.2^+^ T cells determined by FACS. (B) Flow cytometry of sorted GFP^−^CD4^+^CD45.2^+^ T cells before injection. (C) Analysis gate for adoptively transferred CD45.2^+^ T cells on day 0. (D) Absolute numbers of FOXP3^−^GFP^+^CD4^+^CD45.2^+^ T cells (mean±SEM, *n*=3 mice per group).

To look for evidence of conversion in immunocompetent recipients where homeostatic proliferation should not be a confounding factor, 3×10^6^ FACS sorted CD45.2^+^GFP^−^CD4^+^ cells from H2^b^ FOXP3^GFP^-reporter mice were adoptively transferred into congenic CD45.1^+^ H2^b^ recipients, and these mice then received the tolerising anti-CD4/DST protocol (Fig. 7A). The purity of the sorted input GFP^−^ population was >99% (Fig. 7B). Control mice received anti-CD4 mAb alone, DST alone or adoptive transfer only. On day 0, spleens were harvested and analysed by FACS. Transferred cells were readily identified as indicated by the analysis gate (Fig. 7C). As shown in Fig. 7D, the absolute number of CD45.2^+^GFP^+^ cells was >7-fold higher in the anti-CD4+DST group (1.5±0.8×10^3^) compared to the control groups (all <0.2×10^3^), demonstrating that phenotypic conversion of FOXP3^−^ to FOXP3^+^ cells can occur in the presence of a full repertoire of CD4^+^ cells.

Taken together, these data indicate that tolerance induction can drive the direct conversion of FOXP3^−^ precursors into FOXP3^+^ Treg with the capacity to prevent allograft rejection and imply that successful tolerance induction can both increase the proportion of functional alloreactive Treg and by conversion, lead to the depletion of potentially alloreactive effector cells.

### Tolerance induction inhibits Th1 effector cell priming and in vitro cytotoxicity

**Figure 8:**
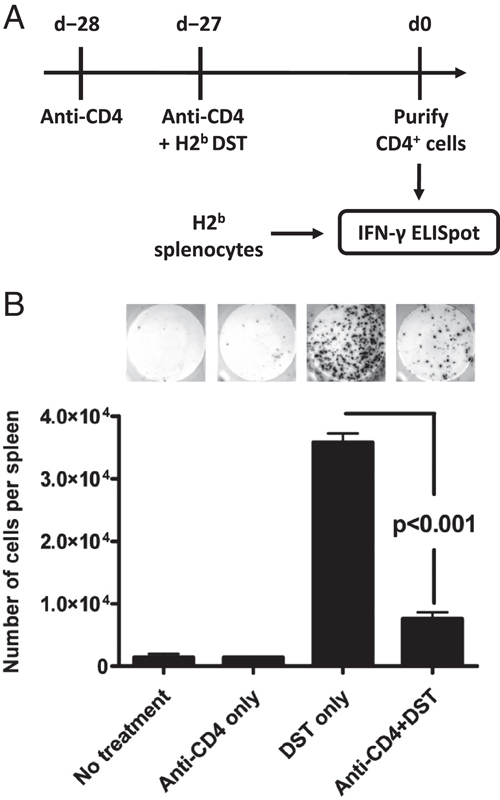
Tolerance induction inhibits CD4^+^ effector cell priming. (A) CBA mice (H2^k^) were pre-treated with 200 μg of YTS177 (anti-CD4) i.v. on days 28 and 27 together with 250 μL whole H2^b^ blood (DST) on day 27. Control mice received anti-CD4 alone, DST alone or no pre-treatment. CD4^+^ cells were purified from spleens harvested on day 0 and stimulated with H2^b+^ splenocytes in an IFN-γ ELISpot. (B) Absolute number of donor-reactive IFN-γ-producing CD4^+^ cells per spleen – pooled cells from four to five mice per group, representative of two independent experiments. Representative ELISpot well images are shown. Statistical analysis using the *t* test.

Naïve CD4^+^ T cells exhibit plasticity following activation and can differentiate into diverse effector populations that in unmodified transplant recipients lead to allograft rejection. We have obtained evidence in two different systems that tolerance induction can result in the emergence of regulatory cells from non-regulatory precursors, but hitherto we have neglected the possible impact of tolerance induction on potential effector cells within the T-cell compartment. Previous work in human renal transplantation has shown a positive correlation between increased frequencies of donor-reactive IFN-γ-secreting T cells and a higher incidence of acute rejection, chronic allograft nephropathy and reduced 1-year allograft function 36–38. Therefore, IFN-γ ELISpot analysis was used to examine the impact of tolerance induction on alloreactive effector T-cell responses. CBA mice were tolerised with the anti-CD4+DST protocol and controls received anti-CD4 mAb alone, DST alone or no treatment (Fig. 8A). Spleens were harvested on day 0 and CD4^+^ cells were challenged with T-cell-depleted H2^b^ donor-strain splenocytes in an IFN-γ ELISpot assay. The frequency of donor-reactive CD4^+^ cells in un-manipulated mice and anti-CD4-only controls was not significantly different from background (Fig. 8B). However, mice that received DST alone showed clear evidence of priming, with large numbers of donor-reactive IFN-γ-secreting CD4^+^ cells in the spleen. In contrast, primed donor-reactive CD4^+^ cells were present at a four-fold lower frequency in tolerised mice compared to DST only controls (7.6±1.0×10^3^ versus 35.8±1.3×10^3^, respectively), indicating that in addition to promoting the development of Treg, a further key effect of tolerance induction in this protocol is an inhibition of alloreactive Th1 priming.

**Figure 9:**
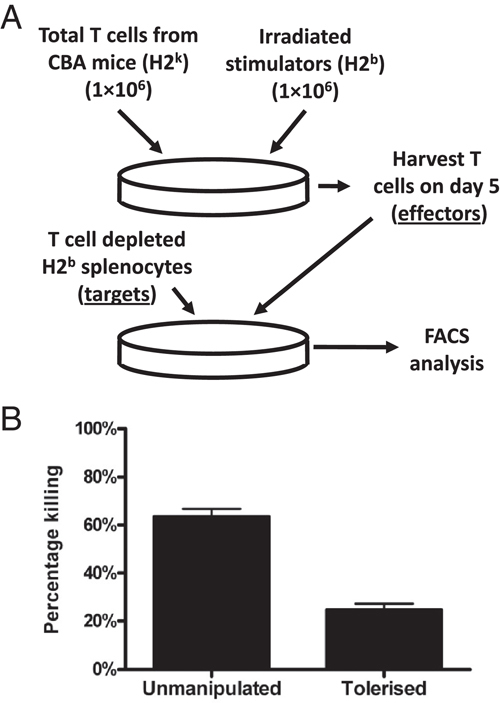
Tolerance induction ameliorates in vitro cytotoxicity. (A) Total T cells from unmanipulated or anti-CD4+DST-treated mice were stimulated in vitro for 5 days with irradiated H2^b^ splenocytes and then harvested (effectors) and cultured for 6 h with H2^b+^ splenocytes (target). The absolute number of live 7AAD^−^K^b+^ target cells was determined by FACS. (B) Percentage killing (normalised to negative control). Data indicate the mean±SD of triplicate tubes and are representative of four independent experiments each using three to four mice per group.

T-cell effector function was also evaluated in this system using an in vitro cytotoxicity assay. To ask whether there is a difference in the ability of T cells from tolerant mice to kill donor strain targets, total T cells from un-manipulated or tolerised mice were purified by negative selection and re-stimulated with irradiated donor (H2^b^) splenocytes (Fig. 9A). On day 5, the T cells were re-isolated and incubated with fresh donor (H2^b^) splenocyte targets. After 6 h, the absolute number of live TCR-β^−^7AAD^−^Kb^+^ target cells was determined by FACS. As illustrated in Fig. 9B, T cells from tolerised mice had significantly impaired cytotoxicity compared to T cells from unmanipulated mice, confirming that tolerance induction has multiple effects on the immune system, both generating alloreactive Treg and inhibiting the development or function of cytotoxic T cells.

## Discussion

Several experimental approaches for the induction of donor-specific transplantation tolerance have been validated in rodents and in many of these models. CD25^+^CD4^+^ Treg play a critical role in graft prolongation. As a result, many groups have focused their attention on the in vivo or ex vivo generation of Treg, for manipulating immune responses to an allograft. Adoptive transfer experiments in the present study show that while CD25^+^CD4^+^ cells from un-manipulated mice suppress allograft rejection at high Treg:Teff ratios, these cells are significantly less effective than CD25^+^CD4^+^ cells from tolerised mice. Importantly, the fact that graft-protective Treg can be generated from CD4^+^ precursors devoid of nTreg underlines the importance of adaptive/induced Treg, but also demonstrates that graft outcome is critically dependent on the balance of graft-protective and graft-destructive T cells. Thus, the data suggest that adaptive alloreactive Treg can arise by both expansion and conversion, and that uncommitted cells can be subverted to become regulatory cells. These data support the view that peripheral T cells have a considerable capacity for plasticity in terms of both loss and acquisition of specific functions. For example, lineage-tracing experiments have shown that under inflammatory conditions it is possible to find in the pancreas of NOD mice a population of ex-Treg that, though once positive for FOXP3 have become FOXP3^−^ 39. Similarly, it has recently been shown that when transferred to T cell-deficient hosts, FOXP3^+^ T cells can accumulate in Peyer's patches of the gut and promote germinal centre formation and B-cell activation. Significantly, the majority of T cells participating in these interactions lose FOXP3 expression 40. Although the cues that drive functional plasticity have not been fully elucidated, it seems quite likely that inflammation and the presence or absence of TGF-β play an important role 41. This seems to be particularly the case with respect to Th17 and induced (adaptive) Treg where fate-switching seems to be dependent on the local availability of TGF-β 42. In this regard, it is interesting to note that the anti-CD4/DST protocol delivers alloantigen via the intravenous route and thus challenges the immune system in the absence of overt inflammation. Thus, one simple possibility is that in the absence of inflammation, plasticity can result in a non-Treg to Treg conversion (Fig. 5 and 7), while in the presence of inflammation, the opposite occurs. A simple distinction in outcome based on the presence or absence of inflammation would have clear physiological benefits in the context of wider immunity by maintaining peripheral regulation of responses to self but also allowing the rapid elaboration of protective immunity following infection where the presence of pathogens is invariably accompanied by pro-inflammatory stimuli 43. In this regard it is interesting to note that in an analogous mouse tolerance induction model, while pre-treatment with an anti-CD154/DST protocol leads to long-term cardiac allograft survival, the addition of TLR9 ligation by co-delivery of exogenous CpG results in acute rejection 44. Thus, the inflammatory context in which alloantigen is first encountered appears to have a significant impact on the eventual immunological outcome.

An additional aspect raised by the current study is that although much of the evidence for T-cell plasticity has been obtained by adoptive transfer of defined populations into lymphopenic or T-cell-depleted hosts, the data in Fig. 7 demonstrate that non-Treg to Treg plasticity can be detected in immunocompetent hosts. This appears to be an important observation with respect to transplantation because it implies that it may be possible to convert naïve alloreactive CD4^+^ T cells into graft-protective Treg in clinical transplant recipients without the need for large-scale T-cell depletion.

Several publications support the observation that uncommitted non-Treg can undergo peripheral conversion into cells that are phenotypically and functionally indistinguishable from naturally occurring Treg 31, 32, 45–47. However, a recent study from Nagahama et al. reported conflicting findings in a minor histocompatibility antigen-mismatched mouse transplantation model 48. In this system, T-cell-deficient BALB/c mice (H2^d^) reconstituted with syngeneic T cells prior to treatment with anti-CD4 mAb (YTS177.9) plus DBA/2 DST accept DBA/2 (H2^d^) skin allografts long term. However, the same tolerance protocol was ineffective when the mice were reconstituted with GITR^−^CD4^+^ T cells rather than total T cells, implying that input Treg are necessary for tolerance induction. The predominant pathway of allorecognition through which graft-protective Treg are generated following tolerance induction is unclear but one possible explanation for the contradiction between the data presented in Fig. 5 (MHC mismatch) and those reported by Nagahama et al. is that conversion of non-Treg precursors during tolerance induction occurs predominantly within the direct rather than the indirect alloreactive T-cell pool.

Although there is evidence that CD8^+^ T cells can be regulated by CD4^+^ adaptive Treg 49, it is likely that to achieve operational tolerance in the wider setting, additional strategies will be required to control CD8 responses, particularly those of CD8 memory cells 50. In this study we demonstrate that total T cells from tolerised mice kill donor-strain cells less efficiently than cells from un-manipulated mice, raising the possibility that in addition to generating CD4^+^ Treg, tolerance induction in this system leads to the functional deletion of alloreactive cytotoxic CD8^+^ cells. Indeed, a previous study of tolerance induction using anti-CD154 mAb+DST provided clear evidence for such a deletion 51. Further support for this possibility is provided by the finding that co-stimulation blockade was significantly less effective at prolonging allograft survival in mice where T-cell resistance to activation-induced cell death was induced by BCL-XL expression, implying that in some situations, deletion of effector T cells can be an important component of tolerance induction 52. Combining tolerogenic therapy with T-cell depletion has been suggested previously as a method of improving the ratio of graft-destructive and graft-protective T cells 52, 53 but large-scale T-cell depletion places patients at increased risk of infection and post-transplant lymphoproliferative disease 54.

Our data demonstrate that alloantigen-dependent tolerance induction has multiple effects on the recipient immune system, and suggest that in the quest for clinical transplantation tolerance, attention should be focused on developing protocols that not only generate graft-protective Treg but also target potential effector cells. Given the low abundance of nTreg, the possibility of generating graft protective Treg in vivo from the much larger pool of alloreactive non-regulatory T cells is very attractive for the induction of tolerance in the clinic.

## Materials and methods

### Animals

CBA.Ca (CBA, H2^k^), C57BL/6 (B6, H2^b^), C57BL/10 (B10, H2^b^) and CBA.rag1^−/−^ (H2^k^, kindly provided by D. Kioussis, NIMR, London, UK) were housed in the BMSU, John Radcliffe Hospital. All procedures complied with the UK Animals (Scientific Procedures) Act, 1986. C57BL/6.45.2 (H2^b^, CD45.2) and DBA/2 (H2^d^) mice were from the Jackson Laboratory, FOXP3^GFP^-reporter mice (H2^b^, CD45.1) mice are as described previously 55.

### Antibodies

The hybridomas YTS169 (anti-CD8) and YTS177.9 (anti-CD4) were kindly provided by Prof. H. Waldmann (Sir William Dunn School of Pathology, Oxford). The hybridomas TIB120 (anti-MHC class II) and PC61 (anti-CD25) were from the ATCC. Conjugated antibodies were from eBioscience (DTA-1, anti-GITR; PC61.5, anti-CD25; H57-597, anti-TCR-β; FJK-16s, anti-FOXP3; GK1.5, anti-CD4) or BD Pharmingen (AF6-88.5, anti-K^b^).

### Tolerance induction protocol

Mice received 200 μg of anti-CD4 mAb (YTS177.9) i.v. on days 28 and 27 and a DST (250 μL of whole donor-strain blood) i.v. on day 27.

### Skin transplantation

Full-thickness tail skin allografts were transplanted onto recipient graft beds. Rejection was defined as complete graft necrosis. Survival was compared using the Log-rank test.

### Heart transplantation

Heterotopic cardiac transplants were performed essentially as described previously 56. Rejection was defined as lack of palpable cardiac contraction and confirmed by laparotomy.

### Cell purification

T cells were purified by magnetic separation as described previously 21 or by flow cytometry (BD FACSAria).

### IFN-γ ELISpot assay

Ninety-six-well MultiScreen plates (Millipore) were coated with capture mAb (AN18, Mabtech). Responder and stimulator cells were incubated for 14 h followed by washing. Detection mAb (biotinylated R4-6A2, Mabtech) was added, incubated for 2 h at room temperature followed by washing. Streptavidin-Alkaline phosphatase (Mabtech,) was added for 1 h. Plates were washed and developing substrate (Mabtech) was added. Spots were enumerated with an AID ViruSpot plate reader and ViruSpot 3.3 software (Autoimmun Diagnostika GmbH, Germany).

### In vitro cytotoxicity assay

T cells from naïve or tolerised mice were stimulated with irradiated donor splenocytes. Graded numbers of T cells from five day 1 cultures were incubated with (H2^b^) targets for 6 h. The cells were stained with 7AAD, anti-TCR-β and anti-K^b^ antibodies. 7AAD^−^TCR-β^−^K^b+^ targets were enumerated by FACS.

## Abbreviations

DST: donor-specific transfusion
GITR: glucocorticoidinduced TNFR-related protein
MST: median survival time
