# Supplementary material for: Induction of transplantation tolerance converts potential effector T cells into graft-protective regulatory T cells
Source: Eur J Immunol. 2010 Dec 8;41(3):726–38. doi: 10.1002/eji.201040509 (PMC3175037; doi:10.1002/eji.201040509)
Supplement: Supplementary file 1 [file eji0041-0726-SD1.pdf]

# European Journal of Immunology


## Supporting Information

## for


## DOI 10.1002/eji.201040509


**Induction of transplantation tolerance converts potential effector T cells into graft-protective regulatory T cells**


Ross S. Francis, Gang Feng, Thanyalak Tha-In, Ian S. Lyons, Kathryn J. Wood and Andrew Bushell


© 2011 WILEY-VCH Verlag GmbH & Co. KGaA, Weinheim 

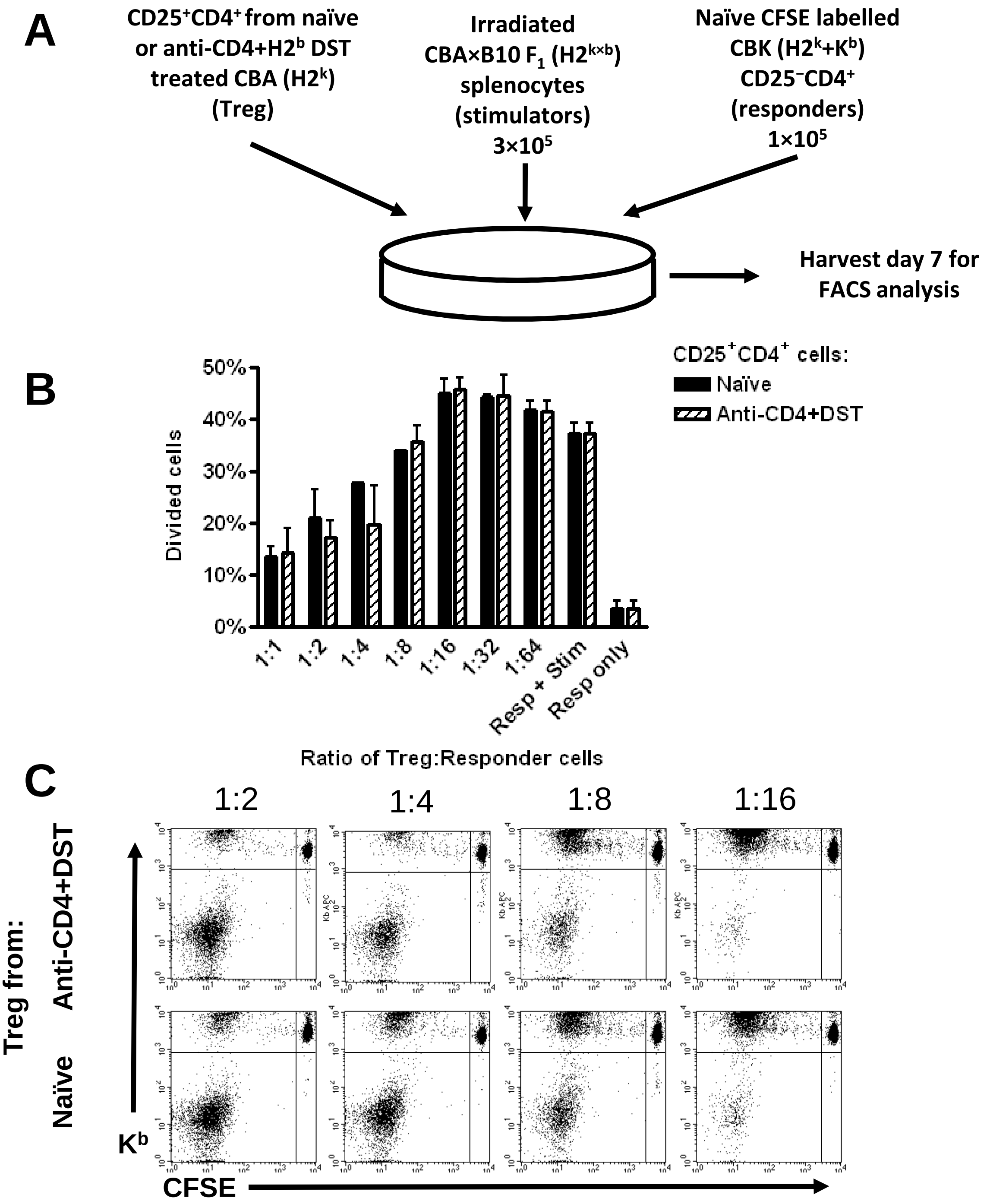


**Supplementary Figure 1: In vitro CFSE-dilution suppression assay**

CD25⁻CD4⁺ cells (responders) were purified from the spleens of naïve CBK mice ($H2^k$ + $K^b$) and labelled with 2µM CFSE. CD25⁺CD4⁺ cells (Treg) were purified from the spleens of naïve or tolerant CBA mice ($H2^k$). Tolerant mice were pre-treated with 200µg anti-CD4 mAb (YTS177) on days −28 and −27 and 250µl B10 ($H2^b$) DST (day −27). Treg from tolerant or naïve mice were titrated into wells containing $1\times10^5$ responders and $2\times10^5$ irradiated CBA×B10 $F_1$ ($H2^{k\times b}$) splenocytes (stimulators). On day 7, cells were harvested and stained with 7AAD, anti-$K^b$ mAb and anti-CD4 mAb. The proportion of responder ($K^{b+}$) cells that had proliferated was calculated by gating on $7AAD^-K^{b+}CD4^+$ cells that had undergone at least one division based on CFSE dilution.

A. Experimental plan

B. Bars indicate the mean (±SD) percentage of divided responders obtained from triplicate wells and are representative of two independent experiments

C. Representative FACS plots

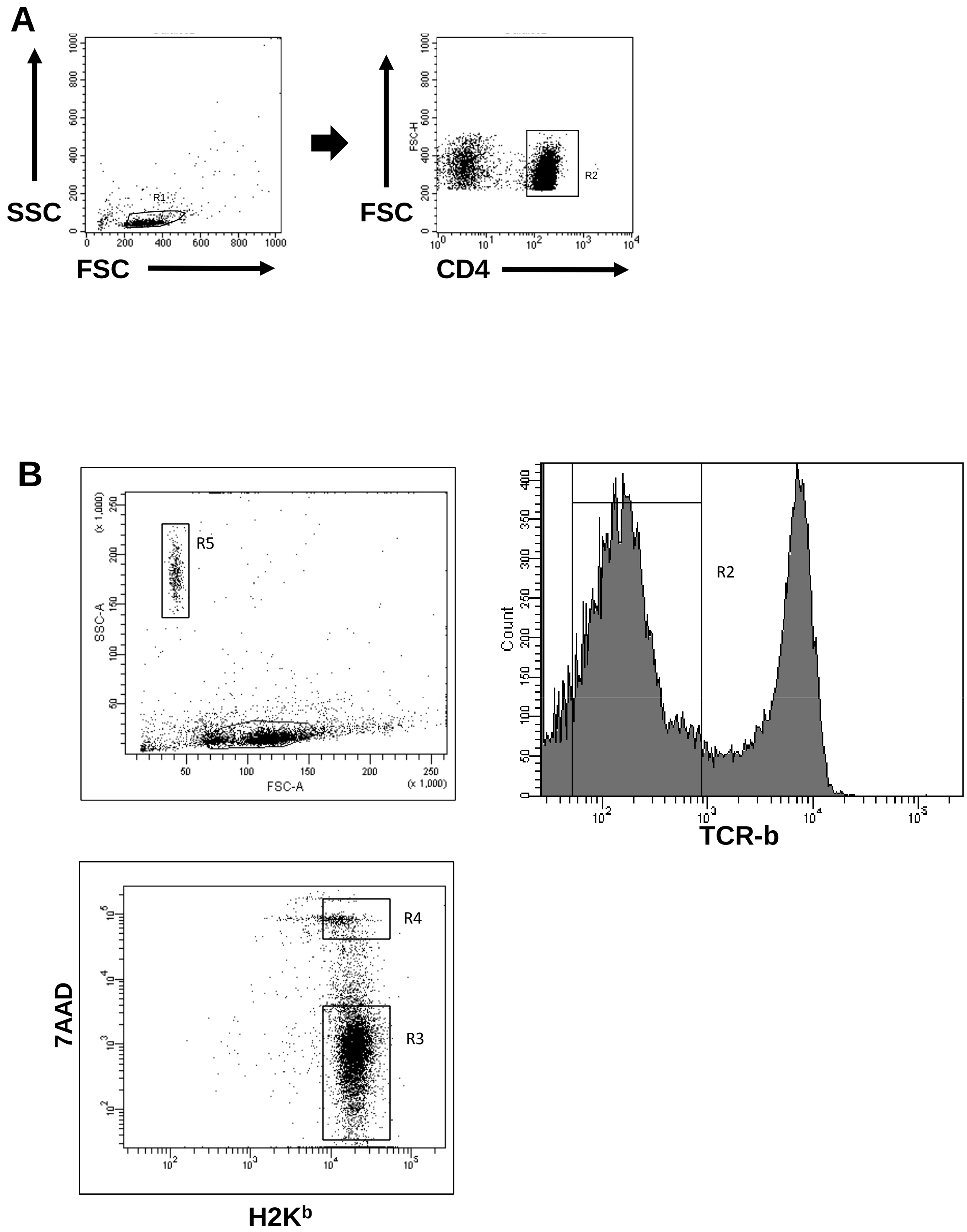


**Supplementary Figure 2: Gating strategies**

A.CBA splenocytes were stained for CD4. FACS plots indicate representative FSC, SSC and CD4 gates used to analyse CD4$^+$ T cell populations throughout this manuscript

B.Killing assay gating strategy: R1 lymphocyte gate; R2 non-T cell targets; R3 viable H2K$^{b+}$ targets; R4 non- viable H2K$^{b+}$ targets; R5 counting beads
